# Anti-BCMA CAR-T therapy for multiple myeloma with extramedullary disease: A case report and review of the literature

**DOI:** 10.1097/MD.0000000000038541

**Published:** 2024-06-28

**Authors:** Huihui Shi, Man Zhang, Yajing Su, Jingwen Liu, Jiayuan Guo, Mingxin Liu, Qiuling Ma

**Affiliations:** a Second Clinical College of Henan University of Traditional Chinese Medicine, Zhengzhou, Henan, China; b Hrain Biotechnology Co. Ltd., Shanghai, P.R. China.

**Keywords:** BCMA CAR-T therapy, extramedullary disease, multiple myeloma

## Abstract

**Introduction:**

Multiple myeloma (MM) with extramedullary disease (EMD) is rare in clinical practice, and B cell maturation antigen (BCMA) CAR-T cell therapy is a novel therapy for hematologic malignancies. Very few reports have been published on the effect of CAR-T-cell therapy in MM with EMD. Here, we report a case of MM with extramedullary lesions treated with BCMA CAR-T therapy.

**Case presentation:**

A 66-year-old female patient presented to our hospital with an enlarged left maxillary gingiva.

**Diagnosis:**

Diagnosis of indolent MM stage III (DS staging) and stage III (ISS and R ISS) with extramedullary lesions.

**Intervention:**

The patient underwent a clinical trial of humanized anti-BCMA CAR T cell therapy.

**Results:**

Symptoms improved; left gingival hyperplasia and swelling resolved; left buccal mass resolved; and neck and submandibular masses resolved. Pathological examination of the exfoliated masses showed necrotic tissue.

**Conclusion:**

MM with extramedullary lesions often has limited treatment options, and traditional chemotherapy methods are ineffective; however, BCMA CAR-T cell therapy can significantly improve the symptoms of extramedullary lesions in MM.

## 1. Introduction

Multiple myeloma (MM) is a malignant tumor caused by the abnormal clonal proliferation of plasma cells, often accompanied by multiple osteolytic pathological changes, anemia, kidney damage, and hypercalcemia. It accounts for >10% of all hematologic cancers.^[1]^ An estimated 34,920 people in the United States and 588,161 people worldwide are diagnosed with MM each year, and this number is increasing each year.^[2]^ Normally, plasma cell proliferation in MM is restricted to the bone marrow, but in rare cases, it can involve other tissues and organs, such as soft tissues, muscles, the central nervous system, and lymph nodes, which is called extramedullary disease (EMD), which can occur at the beginning of the disease or as the disease progresses. In recent years, studies have shown a significant increase in the incidence of EMD.^[3]^ Patients with extramedullary multiple myeloma (EMM) have significantly lower median and 3- and 5-year survival rates than those without extramedullary lesions. This indicates that these patients have a poor prognosis, and most have a low response rate to conventional chemotherapy regimens.^[4]^ Over the past half-century, breakthroughs have been made in the treatment of MM, including therapies such as proteasome inhibitors, immunomodulators, and monoclonal antibodies, which have brought MM treatment into the era of targeted immunotherapy.^[5]^ However, the efficacy of these immunotherapies in the treatment of MM with extramedullary lesions is unsatisfactory, with a short duration of efficacy, and the majority of patients eventually die from disease relapse.^[6]^ Therefore, there is an urgent need for novel therapies to improve clinical outcomes and prognosis in patients with MM with extramedullary involvement. Anti-B cell maturation antigen (BCMA) chimeric antigen receptor (CAR) T-cell therapy is a novel precision-targeted therapy. BCMA is a member of the tumor necrosis factor receptor superfamily, which is expressed predominantly on mature B cells and plasma cells and plays a role in B cell development, with its 2 ligands being the B cell activating factor and the proliferation inducing ligand APRIL. Therefore, BCMA is expressed on the surface of almost all myeloma cells, making it a suitable target antigen for CAR-T therapy.^[7]^ The CAR is a 3-part protein consisting of a single-chain variable region that recognizes tumor antigens, a linker region and an intracellular co-stimulatory molecule.^[8–10]^ T cells are genetically modified in vitro to express CAR, then expanded and infused back into the patient body to play an anti-cancer role,^[11,12]^ which has been optimized and improved in recent years to achieve good results in hematological oncology treatment.^[13,14]^ However, reports of extramedullary lesions in MM are still limited. To discuss the clinical characteristics and treatment strategies of extramedullary lesions of MM, which can serve as a reference for clinical management, we analyzed the clinical data of a patient with extramedullary lesions of MM and reviewed previous literature.

## 2. Case presentation

On March 17, 2021, a 66-year-old woman presented to the hospital with swelling in both feet. Blood tests showed a white blood cell (WBC) count of 2.58 × 10^9^/L, hemoglobin (Hb) of 71 g/L, platelet count (PLT) of 80 × 10^9^/L, uric acid of 489umol/L, C-reactive protein of 23.1 mg/L, and glutamine transaminase of 40U/L. After symptomatic treatment, the Hb and PLT levels continued to decrease. On April 23, 2021, biochemical immunoglobulin G 3.394 g/L, immunoglobulin A 0.047 g/L, immunoglobulin M 0.022 g/L, and serum β2microglobulin 7.29 mg/L were checked. CT showed multiple abnormal signals in the swollen areas of both feet. Morphological examination of the bone marrow cells revealed 81.5% immature plasma cells. Flow cytometry detected 31.2% of abnormal plasma cells with the following immunophenotypes CD38++, CD138+, CD81+, CD19−, CD56−, CD20+, CD13+, CD117−, CD200−, CD28−. Fluorescence in situ hybridization (FISH) of the bone marrow indicated 58% positivity for CCND1/IGH fusion, IGH breaks, and D13S319 (13q-) gene deletion. The 4 free light chain tests (blood + urine) showed sFKAP blood free kappa light chain 6.88 mg/L, sFLAM blood free lambda light chain 6.84 mg/L, srFLC blood free light chain ratio 1.006, urFLC urinary free kappa/urinary free lambda 1.972, uFKAP urinary free kappa light chain 9. 86 mg/L, uFLAM urinary free kappa light chain 9.86 mg/L, and uFLAM urinary free kappa light chain 9. L, uFLAM urinary free lambda light chain 5.00 mg/L, and sdFLC blood free light chain difference 0.04. The patient was diagnosed with “multiple myeloma.” The patient family refused chemotherapy and the patient was discharged.

On January 17, 2022, the blood count was: WBC: 1.78 × 10^9^/L, Hb concentration: 57 g/L, PLT: 4 × 10^9^/L. Morphological examination of the bone marrow cells revealed 93.5% immature plasma cells. Flow cytometry identified 88.96% of the cells (nucleated cells) as abnormal plasma cells. They partially expressed CD38, CD138, CD20, CD56 and did not express cLambda, cKappa, CD19, CD200, CD81, CD28, and CD27. Marrow karyotype analysis: clonal abnormalities occurred −X,+4,?t (8; 12), add (8q),+9, der (10),t (11; 14), der (13; 21),-14,+der (14), ?der (15). Imaging: Decreased height of thoracic 12, lumbar 2 and 4, and abnormal vertebral signals were considered pathological fractures (thoracic 12 and lumbar 2 lesions de novo). Multiple abnormal low-signal shadows in the 1st and 5th lumbar vertebrae, sacrum, and left ilium with sclerotic bone changes MM? Osteoporosis with multiple bone destruction is consistent with the changes in MM. No monoclonal immunoglobulin bands were detected by electrophoresis of serum proteins or immunofixation electrophoresis. Diagnosed with “non-secretory multiple myeloma stage III (DS) stage III (ISS and R ISS).” The patient received IRD chemotherapy (ixazomib, lenalidomide, and dexamethasone), and was discharged after improvement.

**Figure 1.**
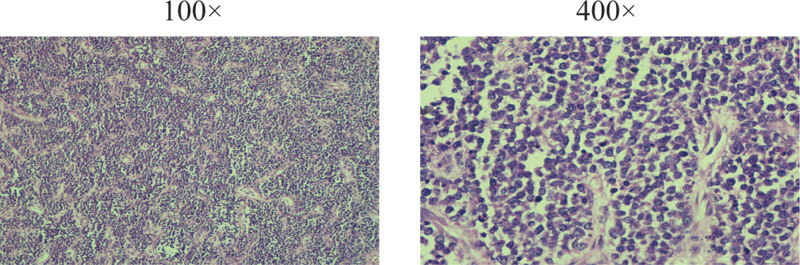
HE staining of the right maxillary gingival mass.

**Figure 2.**
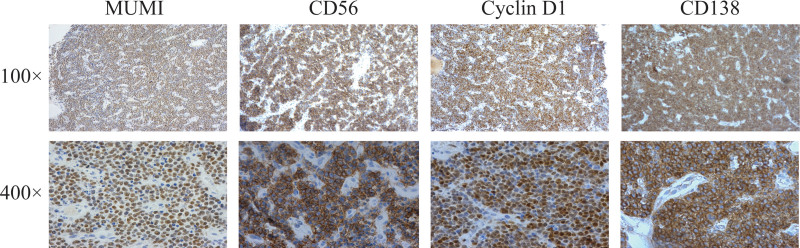
Immunohistochemical results of right maxillary gingiva.

**Figure 3.**
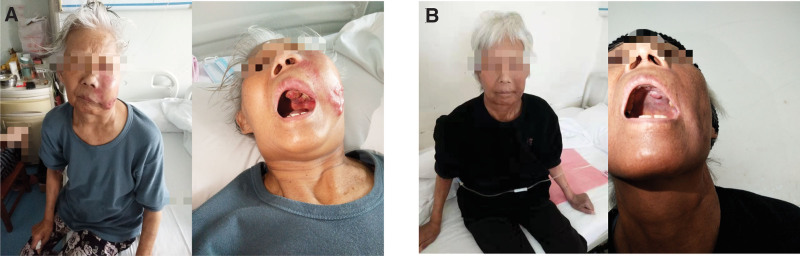
Left maxillary gingival mass. The left maxillary gingival mass subsided after BCMA CAR-T cell therapy. AB: the mass was significantly enlarged before treatment; CD: the mass subsided after treatment. BCMA = B cell maturation antigen.

**Figure 4.**
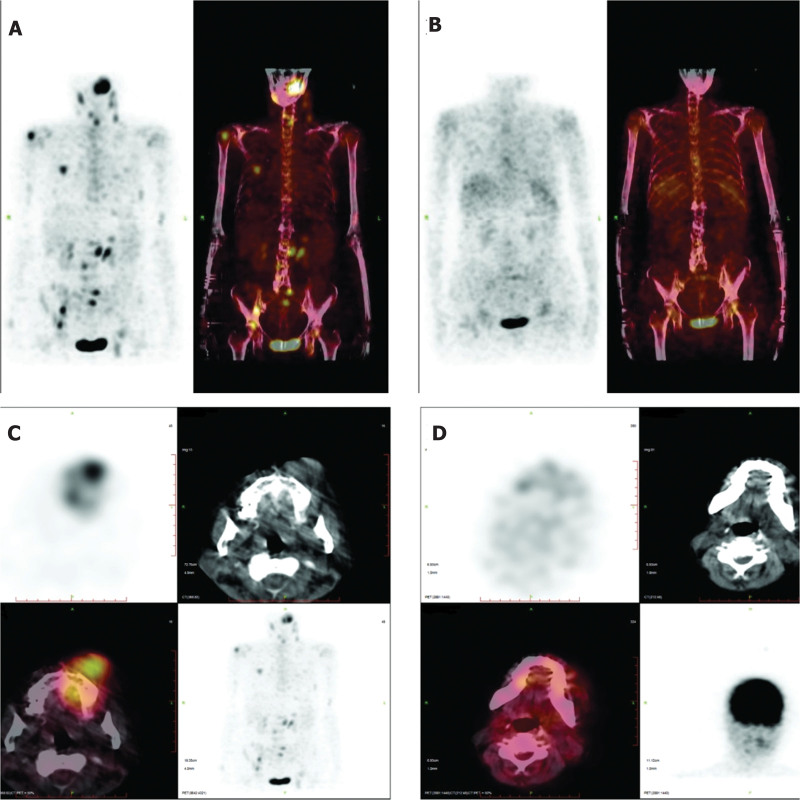
SPECT/CT report before and after treatment. (A) Pretreatment whole-body SPECT/CT report; (B) Post-treatment whole-body SPECT/CT report; (C) pretreatment localized SPECT/CT report; (D) Post-treatment localized SPECT/CT report.

In April 2022, the patient had a lump growing in the right maxillary gingiva, about the size of a green bean, without any obvious trigger, pain, or other discomfort, so he did not pay attention to it. The lumps gradually increased in size. Gingival tissue biopsy showed diffuse infiltration of plasma cell-like cells (Fig. 1). Immunohistochemistry: MUM1(partly +); CD56 (+); cyclin D1 (partly +); CD138 (mostly +); bcl-2 (focally +); Ki-67 (dense areas about 75%–80%+); CD10 (−); Bcl-6 (−); CD20 (−); CD79α (−); CD35(−); CD3 (−); CD21 (−) (Fig. 2). Morphological examination of the bone marrow cells revealed 32.5% immature plasma cells. Flow cytometry detection of minimal residual disease (MRD) was 4.92%. indicated myeloma infiltrating the gingiva. On May 21, 2022, the patient received a PAD chemotherapy regimen (Bortezomib, Adriamycin and Dexamethasone), the gingival tumor lesions were significantly reduced, and the blood counts recovered. One month later, the patient developed gingival hyperplasia and swelling on the left side of the teeth. Bone marrow cytology was performed, and hyperplasia was active, and the percentage of plasma cells was increased to approximately 70%, with predominantly young plasma. MRD:72.89%. Bone marrow cell chromosomes: karyotype description 49 to 50, el (1) (p31), der (1) (q12),7,8, dd (8) (q43),9,t (11; 14), (q23; q32), del (18) (q21),20, el (22) (q12)inc (cp10). Consider the following PD condition: Bortezomib, pomalidomide, dexamethasone, and liposomal doxorubicin were administered on June 29, 2022. The patient was discharged from the hospital after her blood count recovered and her condition improved. On August 13, 2022, the patient was admitted to our hospital with “multiple myeloma” due to swelling and hyperplasia of the left side of the gums. Examination showed marked gingival hyperplasia and swelling on the left side (Fig. 3A and B). Blood test: WBC:2.2 × 10^9^/L, RBC:3.06 × 10^12^/L, Hb:86g/L, PLT:156 × 10^9^/L, neutrophil count1.7 × 10^9^/L, RET:2.7%. Bone marrow images showed an increased percentage of lymphocytes in 5.0% of plasma cells. Flow cytometry showed approximately 1.0% plasma cells with immunophenotypes of CD38++, CD138++, CD19−, CD56−, CD33−, and CD20+, and unremarkable restricted expression of intracellular immunoglobulin kappa and lambda light chains. Bone marrow biopsy revealed hyperproliferation of nucleated cells (approximately 90% of the hematopoietic area), diffuse proliferation of plasma cells, few lymphocytes, and few granulocytes, erythrocytes, and megakaryocytes. Serum light chain: serum kappa light chain 0.72 g/L, serum lambda light chain <0.50 g/L; serum free light chain combination: serum free kappa light chain 6.30 mg/L, serum free lambda light chain 4.23 mg/L, serum free kappa/free lambda 1. 4894Ratio; urine free lambda light chain 184 mg/L, urine free lambda light chain 32.50 mg/L, urine free kappa/free lambda, 5.6615. Urine benzoyl protein electrophoresis, urine protein electrophoresis, serum immunofixation electrophoresis, and serum protein electrophoresis did not yield abnormal monoclonal bands. The SPECT/CT report showed metabolically active MM throughout the body, a metabolically active left gingival soft tissue mass, metabolically active multiple enlarged lymph nodes in the cervical and supraclavicular regions of the neck bilaterally (Fig. 4A and C), and compression fractures of the 12th thoracic vertebra and the 1st lumbar vertebra. FISH showed deletion of RB1 (13q14), amplification of CKS1B (1q21) and CDKN2C (1q32.3), and rearrangement of the IGH gene. Molecular pathology revealed mutations in the NARS gene with a frequency of 2.5% (2935X), TP53 gene with a frequency of 7.8% (2030X), and CCND1 gene with a frequency of 4.8% (1599X). Chromosome karyotyping: karyotype:50 < 2n>, XX,+1, add (1) (p13), add (1) (q21),+6, add (6) (q25),+7, −8, add (8) (q24.1),+9, −10, −10, −11, add (12) (p13),+13, del (13) (q14), der (14)t (11; 14) (q13; q32) × 2, −22,+mar1,+mar2,+mar3,+mar4[3]/46,XX[17]. Suggestive Myeloma Invasion.

**Figure 5.**
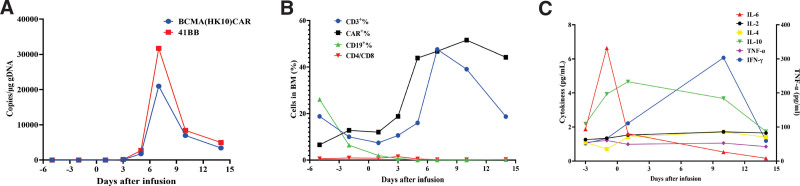
Clinical response after infusion of CAR-T cells. (A) Changes in vector copy number of BCMA CAR after infusion. (B) Proportion of CAR-T cells in peripheral blood. (C) Serum cytokines levels and inflammatory markers were measured at the indicated time points after CAR-T infusion. BCMA = B cell maturation antigen.

Chemotherapy with the VPD regimen (bortezomib + pomalidomide capsule + dexamethasone + daratumumab) was administered between August 18 and August 28, 2022. On August 30, 2022, the patient left gingival hyperplasia and swelling worsened slightly and the left cheek mass increased in size, accompanied by marked tenderness and pain. The patient was enrolled in a clinical trial of anti-BCMA CAR-T cell therapy (ClinicalTrials.gov number, NCT04003168). On September 24, 2022, fludarabine 40 mg and CTX 400 mg were prescribed for lymphodepletion precondition. On September 29, 2022. Human-derived anti-BCMA CAR-T cells from Shanghai Hrain Biotechnology Co., Ltd. were infused at a dose of 9 × 10^6^ CAR-T cells/kg. The vector copy number of BCMA CAR reached its peak at day 7, which was 20976.5 copies/μg. The proportion of CAR-T cells in peripheral blood CD3 cells also reached its peak on day 10, which was 51.6% (Fig. 5A and B). After CAR-T-cell infusion, the level of interleukin-6 (IL-6) gradually decreased, reaching a plateau on day 14 after infusion. The levels of IL-2, IL-4, and interferon-gamma (IFN-gamma) also gradually increased, reaching a peak on day 10 and then decreasing, and IL-10 peaked at the time of CAR-T-cell expansion (Fig. 5C). During treatment, the patient experienced grade 3 cytokine release syndrome (CRS) and other adverse events including fever with neutropenia, anemia, thrombocytopenia, decreased WBC count, nausea, sinus tachycardia, heart failure, pulmonary infection, and delirium, which resolved with symptomatic treatment.

**Figure 6.**
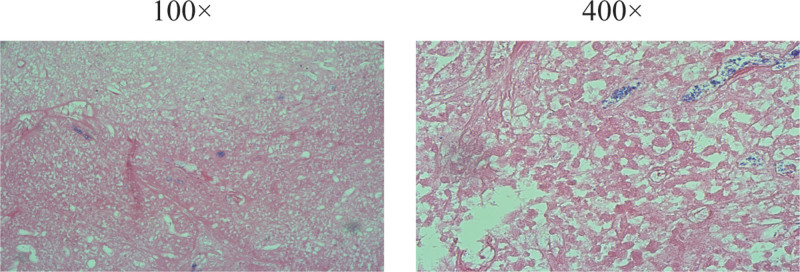
HE staining of the left detached gingival tissue.

At week 2 post-infusion, the bone marrow was evaluated. Myeloproliferative hyperplasia was greatly attenuated, no significant plasma cell-associated quantitative or immunophenotypic abnormalities were detected by flow cytometry, and the patient left gingival hyperplasia and swelling, left cheek mass, and neck and submandibular masses decreased compared to the previous period, achieving PR. In the third week after the transfusion, there was a mass of approximately 1.0*1.0 cm in the left palate, which was locally ulcerated and detached. Bone marrow imaging 4 weeks post-transfusion showed severe hyperproliferative myelopoiesis, and no significant plasma cell-related quantitative or immunophenotypic abnormalities were detected by bone marrow flow. FISH did not detect IGH, TP53/CEP, CKS1B (1q21), or RB1 (13q14) deletions. Bone marrow biopsy showed that the degree of proliferation of nucleated cells in the bone marrow was approximately normal (approximately 30% of the hematopoietic area), the granulocyte/erythrocyte ratio was approximately normal, the granulocyte lineage was dominated by more mature-stage cells, naïve cells were scattered and rare, and lymphocytes were scattered. Immunohistochemistry showed occasional CD138 (+), к (+); λ (+). There were no abnormal monoclonal bands on urine choroidal protein electrophoresis, urine protein electrophoresis, serum immunofixation electrophoresis, or serum protein electrophoresis. Bone marrow chromosome 46, XX [20]. No mutations were found during the follow-up of NGS-positive loci in hematological tumors. The SPECT/CT report at week 6 after reinfusion showed that most of the activity of the systemic osteoprogenitor lesions had disappeared, and the soft tissue mass around the left maxillary alveolar bone and lymph nodes in the left supraclavicular region had become significantly smaller and less active (Fig. 4B and D). The patient left gingival hyperplasia and swelling and left cheek mass had significantly decreased, and the neck and submandibular masses had resolved. In the 8th week after infusion, the patient left gingival mass had resolved hyperplasia, and a soybean-sized mass was seen in the palate with a whitish color and no tenderness. The left cheek mass had resolved, and the neck and submandibular mass had resolved (Fig. 3C and D). Histopathological examination of the excised tumor revealed marked reduction in tumor hyperplasia (Fig. 6). The patient was discharged from the hospital.

## 3. Discussion

We present the case of a patient initially diagnosed with MM with a rare complex karyotype, abnormalities in the IGH, RB1, and D13S319 (13q-) genes, and a high tumor burden. After chemotherapy with proteasome inhibitors (e.g., ixazomib) and immunomodulators (e.g., lenalidomide), the disease continued to progress, with the appearance of a mass in the left maxillary gingiva that continued to grow, metastases in the gingival tissue and bone, and biopsy findings of a plasmacytoma that was ineffective with bortezomib and adriamycin chemotherapy. Therefore, the patient received human anti-BCMA CAR-T cell therapy, and her outcome was assessed as CR at 2 months. This case demonstrates that human anti-BCMA CAR-T cell therapy is effective in the treatment of MM with EMD. Although grade 3 CRS and other adverse events occurred during the treatment, they were manageable.

EMM is a relatively rare subtype of myeloma, and its presentation, progression, and treatment are specific compared to those of intramedullary MM. The pathogenesis of EMM remains unclear and may be related to impaired homing of malignant plasma cells, increased invasiveness, cytogenetic variants, changes in the tumor microenvironment, and the ability of plasma cells to proliferate malignantly independently of the bone marrow microenvironment.^[15]^ If the disease recurs, the survival of patients with EMM is significantly reduced, and the prognosis is poorer due to resistance to conventional treatment. Therefore, some experts believe that EMM should be considered high-risk MM and treated accordingly.^[16]^ Thalidomide is a first-generation immunomodulator, with limited treatment options for EMM. Bladé et al^[17]^ reported that EMM patients did not respond to thalidomide compared with other patients with MM. Lenalidomide, an analog of thalidomide, has been shown to be effective in treating patients with EMM, which may be related to the potent cytotoxic and immunomodulatory effects of lenalidomide.^[18]^ However, due to the high incidence of treatment-related adverse events, such as myelosuppression, neuropathy, and thrombotic events, this regimen can be difficult to tolerate, limiting its widespread clinical use.^[19]^ In contrast, bortezomib, a synthetic dipeptide borate that inhibits the enzymatic action of pancreatic lactase and promotes apoptosis in myeloma cells, appears to be more effective, reversible and selective in patients with EMM and is currently recommended as first-line treatment for patients with EMM. A previous study enrolled 207 patients with EMM, 70 of whom had extramedullary lesions. The results showed that the rate of disease progression during induction was higher in patients with EMM than in those without; therefore, the ability of bortezomib to overcome the poor prognostic value of EMM requires further investigation.^[20]^ In recent years, immunotherapy using CAR-T cells has brought new hope for the treatment of hematological malignancies, including MM.^[21]^ CAR-T immunotherapy involves extracting T cells from a patient blood and genetically modifying them to enhance their ability to target and kill tumor cells. T cells are cultured and expanded in vitro in large numbers and then infused into patients to multiply and ultimately recognize and kill tumor cells, providing a new therapeutic approach for EMM patients.^[22]^ In the first human clinical trial of BCMA CAR T therapy in 16 MM patients, CAR-BCMA T cells were found to be highly active against relapsed/refractory MM (RRMM), with an objective remission rate (ORR) of 81%, and 10 of 16 cases (63%) achieved VGPR or CR.^[23]^ In addition, Xu et al^[24]^ showed that a clinical trial of a dual epitope-targeted CAR-T against BCMA (LCAR-B17M) was conducted in 38 RRMM cases with an ORR of 88.2%, including 13 complete remissions (sCR) and 2 VGPRs, but few efficacy studies have been reported on the efficacy of human anti-BCMA CAR T cell therapy for the treatment of patients with EMD. From a recent limited review of the literature, CAR-T cell therapy appears to be effective in this setting, but the rarity of the disease and lack of prospective trials make it difficult to generate reliable data.^[25]^ In the future, subgroup analyses in large prospective trials focusing on EMM are strongly recommended to help identify the optimal strategies.

In the case reported here, anti-BCMA CAR-T cell therapy significantly improved the clinical symptoms of a patient with MM associated with extramedullary lesions. The patient received an infusion of human anti-BCMA CAR-T cells at a dose of 9 × 10^6^ cells/kg. The peak copy number of BCMA CAR was 20,976.5 copies/μg on the seventh day of infusion. The concentrations of IL-2, IL-4, and γ-interferon (IFN-γ) also gradually increased, peaking on the tenth day and then declining, and IL-2 and IL-10 peaked at the time of CAR-T cell expansion. Ten peaked at the time of CAR-T cell expansion. The level of interleukin-6 (IL-6) gradually stabilized on day 14 after infusion. The SPECT/CT scan at week 6 after infusion showed that most of the systemic osteoprogenitor foci had disappeared, and the soft tissue mass around the left maxillary alveolar bone and lymph nodes in the left supraclavicular region were significantly smaller and had disappeared. In this approach, T lymphocytes are genetically engineered to express a CAR that efficiently directs T cells to recognize and eliminate specific surface antigens on malignant tumor cells in an HLA-independent manner.^[26]^ Therefore, human-derived anti-BCMA CAR-T cell therapy may enhance the proliferation and efficacy of these cells. However, the use of human-derived anti-BCMA CAR-T cell therapy in patients with MM with extramedullary lesions needs to be validated in more rigorous clinical trials.

Despite the promising efficacy of CAR-T therapy for the treatment of EMM, challenges related to its safety and efficacy still need to be overcome, particularly the toxicity of CAR-T cell therapy after administration. In most published studies on this topic, anti-BCMA CAR-T therapy in patients with EMM has been associated with some adverse events. In this case, the patient experienced grade 3 adverse events, including fever with neutropenia, anemia, thrombocytopenia, decreased WBC count, nausea, sinus tachycardia, heart failure, pulmonary infection, and delirium, mainly due to the CRS^[27,28]^ and immune effector cell-associated neurotoxicity syndrome.^[29–31]^ The incidence of these adverse events depends on some factors, including disease characteristics, CAR structure, tumor load, and CAR-T cell dose^[32]^; therefore, patients should be monitored in the hospital before CAR-T cell infusion and for at least 9 days after cell infusion, with close monitoring of peripheral blood images and active symptomatic treatment.^[33]^ In addition, the design of constructs with transient CAR expression, drug-induced switching, and suicide-switching mechanisms can help reduce the toxicity of CAR-T cell-derived therapies.^[34]^ As the number of clinical trials with CAR-T cells has increased, CAR-T cell-related adverse events are being recognized and are generally manageable.

In conclusion, anti-BCMA CAR-T cell treatment for patients with EMD has unlimited potential, but its hematological toxicity and potential adverse effects may be greater than those in non-EMD patients, and the duration and depth of remission are limited. Further studies are needed to reduce toxicity and prolong patient survival by combining CAR-T cell treatment with other new drugs or stem cell transplantation.

## Author contributions

**Conceptualization:** Huihui Shi, Mingxin Liu, Qiuling Ma.

**Data curation:** Huihui Shi, Man Zhang, Yajing Su, Jingwen Liu, Jiayuan Guo.

**Investigation:** Yajing Su.

**Methodology:** Huihui Shi, Jingwen Liu, Jiayuan Guo.

**Visualization:** Huihui Shi, Man Zhang.

**Writing – original draft:** Huihui Shi.

**Writing – review & editing:** Huihui Shi, Man Zhang, Yajing Su, Jingwen Liu, Jiayuan Guo, Mingxin Liu, Qiuling Ma.
